# Lung adenocarcinoma presenting as obstructive jaundice: a case report and review of literature

**DOI:** 10.1186/1477-7819-6-120

**Published:** 2008-11-11

**Authors:** Stephanos Pericleous, Samrat Mukherjee, Robert R Hutchins

**Affiliations:** 1 Department of HPB Surgery, Imperial College, Hammersmith Hospital campus, Du Cane Road, London, UK; 2 Department of HPB Surgery, Royal London Hospital, Whitechapel, London, UK

## Abstract

**Background:**

Lung cancer is known to metastasize to the pancreas with several case reports found in the literature, however, most patients are at an advanced stage and receive palliative treatment.

**Case presentation:**

We describe the case of a 56 year old male patient who presented with a picture of obstructive jaundice. Investigations revealed an obstructing lesion in the pancreas and a further lesion in the lung with benign appearances. The patient underwent a pancreatectomy and, unexpectedly, the histology of the resected specimen demonstrated metastatic adenocarcinoma of bronchogenic origin. He was referred to a cardiothoracic team who proceeded to resect the patient's thoracic lesion before administration of adjuvant chemotherapy. The patient was reviewed 18 months post operatively and remains symptom free with no clinical or radiological evidence of recurrence. We were unable to identify any previous case reports (of lung adenocarcinoma) with such a presentation which were ultimately treated with resection of both lesions.

**Conclusion:**

Similar situations are bound to arise again in the future and we believe that this report could demonstrate that there is a case for aggressive surgical management in a highly selected group of patients: those with NSCLC and a synchronous solitary pancreatic deposit.

## Background

That a variety of malignant tumours can metastasise to the pancreas is well documented. Several case reports have reported patients with lung cancer whose clinical presentation was that of obstructive jaundice [1].

Most patients presenting in this manner are at an advanced stage with widespread disease, and are usually managed symptomatically. This generally involves palliative chemotherapy and/or radiotherapy coupled with other measures to relieve the biliary obstruction such as biliary stent insertion. In the few cases where operative intervention is considered, it is usually limited to a biliary bypass to relieve the jaundice.

We describe an unusual presentation where an adenocarcinoma of the lung with a synchronous solitary metastatic deposit in the pancreas (not visible on CT) was treated with operative resection of both lesions. The uniqueness of this case is enhanced by the fact that both lesions were identified preoperatively although their nature was not.

## Case presentation

A 56 year old male lawyer presented to his local hospital complaining of a recent change in his urine colour (to bright orange) and general malaise. The patient suffered from moderate bronchiectasis and asthma for which he took inhalers (fluticasone propionate, salmeterol and ipratropium bromide). He was also known to be hypertensive (controlled on diltiazem) and suffered from severe eczema. He had never been a smoker but his daily consumption of alcohol amounted to 1.5 bottles of wine.

**Figure 1:**
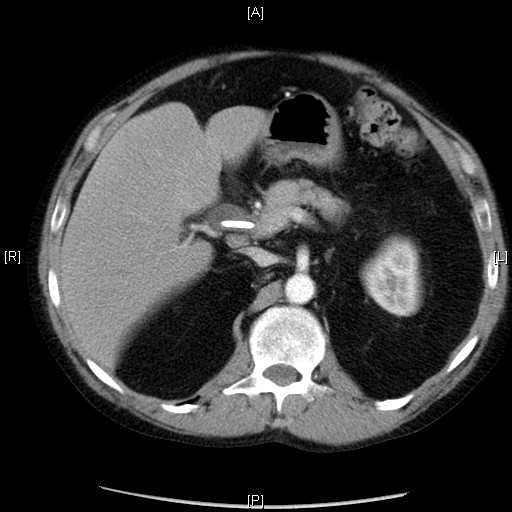
**CT scan abdomen.** Stent visible in bile duct.

Initial workup revealed deranged liver function tests and relevant tumour markers were raised (Ca 19-9 181 kU/l, CEA 25.8 μg/l). A subsequent abdominal ultrasound showed biliary dilatation to the level of the pancreas. This was confirmed on an MRCP. However CT (64 slice fine cut spiral pancreas protocol CT) and MRI examinations failed to reveal any pancreatic mass (figure 1). An ERCP which followed confirmed the lower CBD stricture with features of external compression and a plastic biliary stent was inserted.

**Figure 2:**
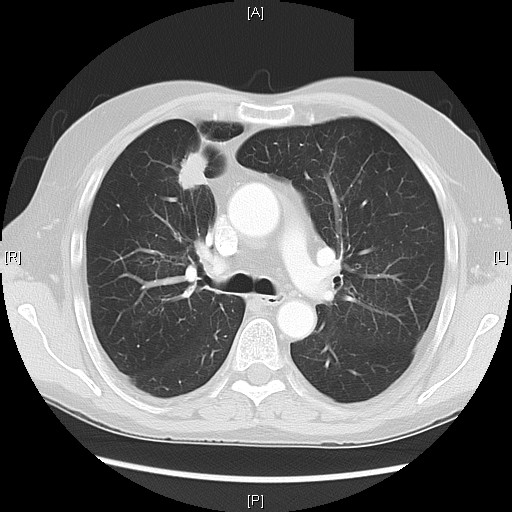
**CT scan chest.** Lesion in the right lung.

**Figure 3:**
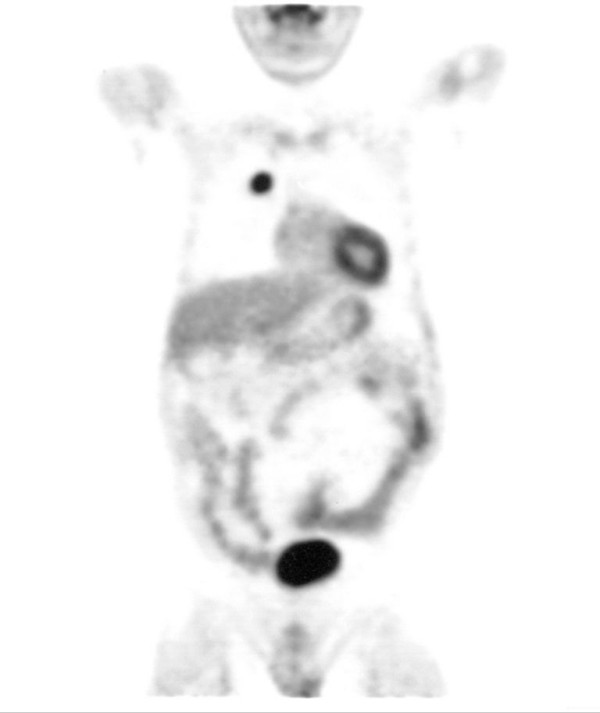
**FDG PET scan.** Lesion in the right lung.

The patient was then referred to our unit for further treatment. The working diagnosis at this stage was a pancreatic tumour and the patient underwent staging with a view to a pancreatic resection. Unusually, as part of the initial workup, the patient had had a CT of his thorax, showing a right lung lesion, thought to be benign, on a background of known chronic respiratory disease (figure 2). A FDG-PET scan was performed to delineate the lung lesion further (figure 3). This scan was reported as positive, thus raising the possibility of:

• A lung primary with pancreatic metastasis

• Synchronous pancreatic and lung primaries

• A pancreatic primary with lung metastasis

CT guided biopsy of the lung lesion was performed, the histology of which showed reactive changes but no evidence of malignancy. As such and in view of the patient's background of respiratory disease the PET scan was interpreted as demonstrating reactive changes. Given the presentation, tumour markers, imaging appearances and biopsy results the working diagnosis remained that of a pancreatic cancer with no evidence of metastatic disease.

**Figure 4:**
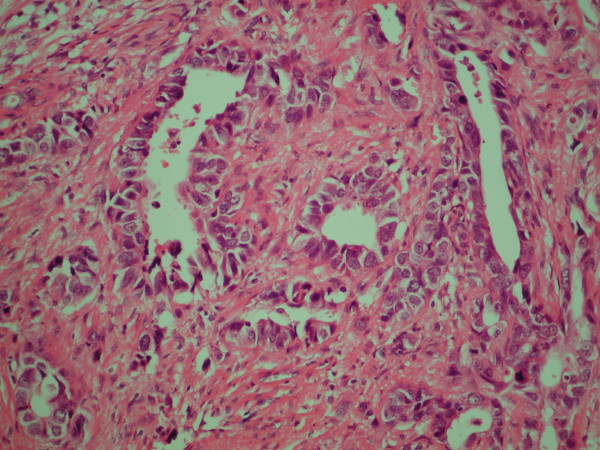
High magnification view of lesion resected from the pancreas (haematoxylin and eosin).

**Figure 5:**
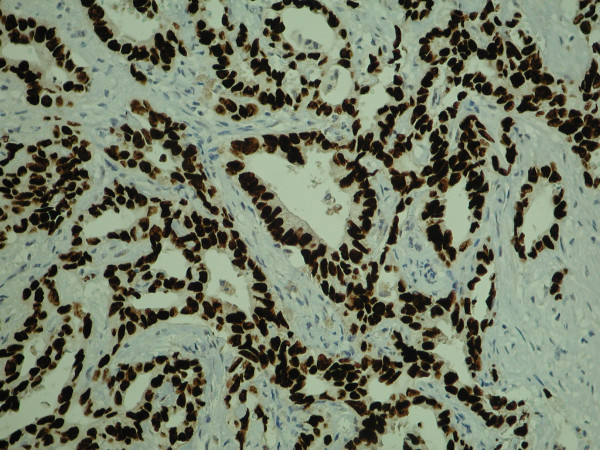
High magnification view of lesion resected from the pancreas (immunohistochemical staining with TTF-1).

The patient proceeded to a pylorus preserving pancreaticoduodenectomy (PPPD). There was no evidence of intra-abdominal spread at laparotomy. The head of the pancreas contained a palpable mass. This was resected in routine fashion. The histology of the resected specimen was a single poorly differentiated adenocarcinoma (figure 4) (11 mm in maximum dimension) staining strongly positive to TTF-1 and CK7 (figure 5), and negative staining for CK20 and PSA. The tumour did not approach any of the resection margins or surfaces. Also, none of the surrounding 16 lymph nodes had any evidence of disease.

In view of the reported immunohistochemical profile, coupled with the identification of a lung lesion, the tumour was interpreted as metastatic adenocarcinoma of bronchial origin rather than as a primary pancreatic lesion. As a result the patient was referred to a thoracic surgeon for consideration of removal of the lung lesion. Six weeks later the patient underwent a mini thoracotomy where a 2 × 3 cm lesion was identified in the medial segment of the upper lobe of the right lung. The segment was removed along with hilar and mediastinal lymph nodes for staging. Histology of this specimen reported a lung adenocarcinoma with complete excision and no lymph node involvement.

Three weeks after his lung resection the patient was started on adjuvant chemotherapy with gemcitabine and carboplatin. This regime was continued for 6 months. The patient was seen eighteen months from presentation. Clinically he remained symptom free and a follow-up CT of his chest and abdomen revealed no evidence of recurrence.

## Discussion

Pancreatic cancer is one of the leading causes of cancer deaths ranking 4th in the US and 6th in Europe [2]. However, little attention is devoted to secondary deposits of other tumours to the pancreas. Retrospective studies on pancreatectomy procedures have reported that metastatic disease represents merely 3% or so of resected malignant pancreatic masses [3,4]. As such they are often mistaken as pancreatic primaries and only recognised for what they truly are in retrospect on histological examination [5]. Some 98% of patients with a malignant process who present with obstructive jaundice will do so as a result of a primary pancreatic cancer [6]. On the other hand, autopsy statistics suggest that the pancreas is a more frequent site for metastatic disease, albeit on a subclinical scale. The incidence of secondary pancreatic tumours is up to 16% of autopsy studies [7], with a wide variation of primary cancers responsible. Patients who present with a clinical picture which relates directly to disease in the pancreas at presentation will tend to do so with the symptoms of obstructive jaundice or pancreatitis [8]. More often than not these patients prove to have advanced disease which is only amenable to palliative treatment.

Lung cancer metastasizes to many sites, but most frequently to bone, the liver and the adrenal glands [9,10]. Approximately one third of patients will present with symptoms relating to extra thoracic spread [10]. The pancreas is considered to be an infrequent target to which lung cancer will metastasize to. Figures are reported in the range of 0–12% [11-13]. The majority of those which do are of SCLC histological subtype [14]. Rarer still, at presentation, is for lung cancer to present with a clinical picture of jaundice due to synchronous metastatic adenocarcinoma [1]. In those cases where it does, this is more likely to be due to widespread hepatic disease than to extrahepatic biliary obstruction [15]. A larger subgroup of patients with lung cancer will develop a metachronous pancreatic metastasis, which will usually be identified on follow-up investigations. One recent case report published in March 2008 reports the first case of lung adenocarcinoma with a metachronous isolated deposit in the pancreas and no evidence of other disease. This case was treated with biliary stenting and palliative chemotherapy [16].

Of secondary deposits discovered in the pancreas, lung cancer makes up (along with renal cell carcinoma, breast and gastric cancer) a high percentage (table 1) [7,17-36]. Indicative published figures are 14.2% (49 of 311 secondary tumours) [7], 17.0% (18 of 108)[18] and 18.2% (4 of 22) [17]. The large majority of cancer patients with metastatic disease to the pancreas are treated with palliative intent as patients usually present with widespread disease. Where surgery is contemplated, it is usually limited to bypass procedures in patients with obstructive jaundice. There have been reports where patients with this presentation have undergone more major procedures such as pancreatic resection[37], but this has tended to be in ignorance of the fact that the aetiology of the obstruction was of metastatic origin, as was in our case. There are several publications advocating the consideration of a pancreatic resection in selected cases. One of these is a literature review by Minni *et al*, where 333 cases with secondary deposits in the pancreas were reviewed. Of these, 234 had treatment information of which 150 (64.1%) underwent pancreatic resections [3]. More than 25 different histologic types are reported 45.0% of which were renal cell, 14.7% lung, 7.5% breast and 6.6% colonic carcinomas. In a series of twelve patients with a variety of different metastatic tumours to the pancreas, Le Borgne *et al *[38], suggest that a more aggressive surgical approach should be considered, especially in patients with metachronous ampullary and pancreatic deposits from renal cell carcinomas, sarcomas and carcinoid tumours. They reported 35% survival rate at 2 years and 17% at 4 years.

**Table 1:** Summary of world literature on pancreatic metastases from lung cancer

**Lung cancer histology subtype**
Small Cell Lung Cancer (22)
Adenocarcinoma^1 ^(4)
Large Cell (2)
Squamous Cell (2)
Anaplastic bronchial (1)
'Lung Cancer'^2 ^(4)
**Presenting symptoms**
Obstructive Jaundice^1 ^(15)
Acute Pancreatitis (13)
No Symptoms^3 ^(5)
Gastrointestinal bleed (1)
Not Available (1)

**Treatment Received**^4^
Palliative Chemotherapy (13)
Biliary stent (8)
Palliative Operation (4)
Best Supportive Care (7)
Pancreatic Resection (6)
Adjuvant Chemotherapy (2)
Exploratory laparotomy (1)
Includes our case. ^2 ^No further information from authors ^3 ^Includes patients who were identified on surveillance. ^4 ^Some patients received more than one treatment.

papers reviewed: [6,8,16,17,19-38,47]

Stage IV NSCLC has a poor prognosis. Median survival with best supportive care is reported as 3.6 months (range, 2.4 to 4.9 months) whilst platinum based chemotherapy regimes increase this statistic to 6.5 months (range, 4.7 to 8.5 months). This patient is alive and disease free 18 months following presentation. It is accepted practice today to consider selected patients with solitary intracranial deposits for resection [39-41]. Also it has been suggested repeatedly that a survival benefit may be achieved by surgical treatment of solitary extracranial spread of NSCLC [42-46]. The experience and information available for the surgical treatment of metastatic disease from the lung exclusively to the pancreas is very limited and few guidelines are available on the appropriate management of such cases. Most series describe treatment which, from the outset had a palliative intent. Hiotis *et al *[47], however, report three cases of patients with metachronous (information from personal correspondence with author) NSCLC metastatic disease to the pancreas who underwent pancreatectomies with curative intent. All patients developed recurrence.

## Conclusion

In the majority of cancers, synchronous presentation generally carries a worse prognosis than a metachronous one. Our case is an example of a synchronous metastatic deposit resected (albeit) inadvertently. However, resection of both lesions has led to long-term disease-free survival. Therefore we believe that this report demonstrates that in selected cases consideration should be given not just to palliation but to potentially curative surgery whether it be synchronous or more likely metachronous presentation of metastatic lung cancer to the pancreas. This is very different from what has been described previously where very few operations with curative intent have been carried out, in particular on patients with NSCLC.

## List of abbreviations

CT: Computed Tomography; MRCP: Magnetic Resonance Cholangiopancreatography; ERCP: Endoscopic Retrograde Cholangiopancreatography; CBD: Common Bile Duct; FDG-PET: Fluorodeoxyglucose – Positron emission tomography; NSCLC: Non-small cell lung carcinoma; TTF-1: Thyroid Transcription Factor-1; PSA: Prostate Specific Antigen; CK7, CK20: Cytokeratin 7, Cytokeratin 20.

## Consent

Written consent was sought and obtained from the patient prior to publication of this article.

## Competing interests

The authors declare that they have no competing interests.

## Authors' contributions

SP operated on the patient, conducted the collection of the data and the literature and conceived the case report. SM was involved in collection of literature and drafting the article. RRH was the principal investigator, operated on the patient collected data and was involved in the drafting of the article.

All the authors have read and approved the final manuscript.
